# Should colorectal cancer screening start at the same age in European countries? Contributions from descriptive epidemiology

**DOI:** 10.1038/sj.bjc.6604488

**Published:** 2008-07-15

**Authors:** H Brenner, M Hoffmeister, U Haug

**Affiliations:** 1 Division of Clinical Epidemiology and Aging Research, German Cancer Research Center, Bergheimer Strasse 20, Heidelberg D-69115, Germany

**Keywords:** colorectal cancer, screening, prevention

## Abstract

We assessed incidence and mortality of colorectal cancer (CRC) at various ages among women and men in 38 European countries. The ages at which defined levels of incidence and mortality were reached varied between 9 and 17 years between countries. This variation requires consideration in the definition of screening guidelines.

Colorectal cancer (CRC) is the third most common cancer and the fourth most common cancer cause of death globally (Parkin *et al*, 2005). In Europe, more than 400 000 new cases and more than 200 000 deaths occur per year (Ferlay *et al*, 2007). On account of its typically slow development, there is a large potential to reduce the burden of the disease by early detection and removal of precancerous lesions or early cancer stages. Various screening examinations, including fecal occult blood testing, sigmoidoscopy and colonoscopy have meanwhile been recommended by expert committees (eg Europe Against Colorectal Cancer, 2007), and nationwide screening programmes are currently being implemented, prepared or under consideration in different European countries. Within the European Union, recommendations on cancer screening typically foresee a defined common starting age (eg Advisory Committee on Cancer Prevention, 2000). However, CRC incidence and mortality strongly vary within Europe. The aim of this study was to assess differences in CRC incidence and mortality within Europe, in view of the potential implications regarding variation of age at screening initiation between countries.

## Materials And Methods

### Analytic strategy

Our analyses are based on the following strategy: We looked at median CRC incidence and mortality among men across Europe at ages 50, 55 and 60, and we determined at what ages these levels of incidence and mortality are reached among men and women in each country. The rationale behind this ‘risk advancement approach’ (Brenner *et al*, 1993) is as follows: CRC incidence and mortality strongly increase with age. The age at which CRC screening becomes meaningful and cost effective depends, among other factors, on CRC incidence and mortality surpassing some minimum threshold in the absence of screening. This threshold, however defined, is reached at various ages in different countries. All of the aforementioned ages have been recommended or are actually used in practise for initiation of CRC screening (Advisory Committee on Cancer Prevention, 2000; Malila *et al*, 2005; Pox *et al*, 2007; West *et al*, 2007).

### Database

Estimates of CRC incidence and mortality rates for age groups 15–44, 45–54, 55–64, and 65+ years were obtained for 38 European countries from the GLOBOCAN 2002 database (Ferlay *et al*, 2004).

### Statistical analyses

In our analyses, we made the following approximations: CRC incidence and mortality at age 40 years were approximated as 0.75 × 3=2.25 times the corresponding rates in the 30-year interval 15–44 years. This approximation is based on the observation that roughly 75% of CRC cases diagnosed in the age range 15–44 years occur in the age interval 35–44 years in populations where more detailed age-specific rates are available, and on the assumption that age interval 35–44 years typically comprises roughly one-third of the population in age interval 15–44 years. CRC incidence and mortality at ages 50 and 60 were assumed to equal the corresponding rates in age groups 45–54 and 55–64 years, respectively. Finally, it was assumed that data on CRC incidence and mortality in men and women in age group 65+ years correspond to the respective levels at the mean age of the male and female population aged 65+ years in each country in the year 2000, which was derived from the United Nations Population Database (United Nations Population Division, 2007).

Based on the assumptions described above, defined levels of CRC incidence and mortality were available for ages 40, 50, 60, and for an age between 72 and 75 years (men) or between 73 and 77 years (women), respectively, with the latter varying across countries. Incidence and mortality at single years of age between these defined levels were approximated by linear interpolation. Sensitivity analyses varying the aforementioned approximations within plausible ranges led only to very minor variation of the results and are therefore not shown in detail.

## Results

**Figure 1:**
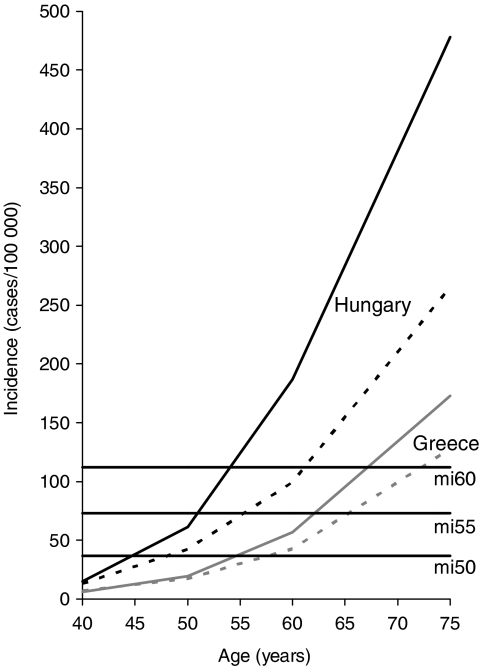
Age-specific incidence of colorectal cancer among men (solid lines) and women (dashed lines) in Hungary (black lines) and Greece (grey lines). The vertical lines indicate the median incidence across European countries among men aged 50 (mi50), 55 (mi55) and 60 years (mi60), respectively.

CRC incidence strongly increased with age in all countries. Estimates of median incidence (mi) across countries among men aged 50, 55 and 60 years were 37, 73, and 112 per 100 000 persons per year, respectively. The age at which these levels were reached among men and women in the different countries, denoted age_mi50_, age_mi55_, and age_mi60_, respectively, varied strongly. For example, among men, age_mi50_ varied between 45 years in Hungary and 55 years in Greece (see Table 1). Similarly, age_mi55_, and age_mi60_ varied between 51 and 62 years, and 54 and 67 years among men in the same countries. As illustrated in Figure 1, this is explained by the much steeper rise in CRC incidence with age in Hungarian men compared with Greek men. Overall, among men in Western Europe, incidence rates were higher, and the various levels of incidence were reached at younger ages compared with men from other European regions. However, there was also substantial variation within regions.

Among women, incidence rates were generally lower compared with men, which implies that specified levels of incidence were reached at higher ages. With few exceptions, between-country variation was similar among women and men, but it was particularly large among older women, reaching a maximum of 17 years (from 60 to 77 years) for age_mi60_.

**Figure 2:**
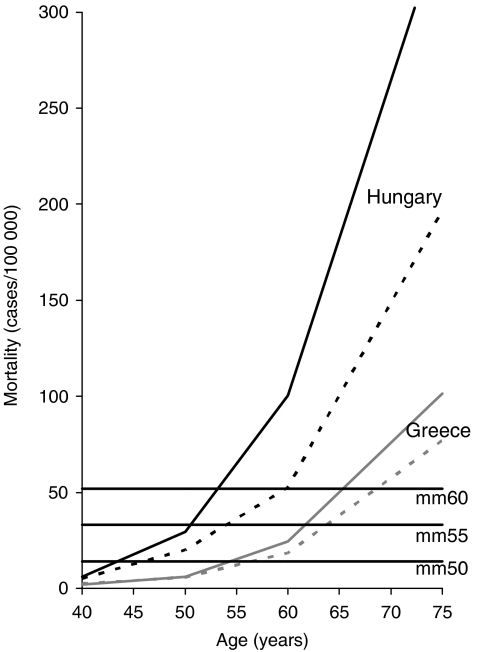
Age-specific mortality of colorectal cancer among men (solid lines) and women (dashed lines) in Hungary (black lines) and Greece (grey lines). The vertical lines indicate the median mortality across European countries among men aged 50 (mm50), 55 (mm55) and 60 years (mm60), respectively.

CRC mortality likewise strongly increased with age in all countries. Estimates of median mortality (mm) across countries among men aged 50, 55 and 60 years were 14, 33, and 52 per 100 000 persons per year, respectively. The age at which these levels were reached among men and women in the different countries, denoted age_mm50_, age_mm55_, and age_mm60_, respectively, again showed strong between-country variation. The lowest and highest levels of these ages (resulting from highest and lowest levels of mortality) were again mostly seen for Hungary and Greece, respectively (see Table 2 and Figure 2). Age_mm50_, age_mm55_, and age_mm60_ varied up to 12 years between countries among both women and men. Again, these ages tended to be higher among women (ranges across countries: 45–57, 54–64, and 60–69 years, respectively) than among men (ranges across countries: 43–54, 51–62, and 53–65 years, respectively). In Eastern countries, mortality was generally higher, and levels of age_mm50_, age_mm55_, and age_mm60_ were generally lower than in countries from other parts of Europe.

## Discussion

Our comparative analyses of age- and sex-specific CRC incidence and mortality in 38 European countries indicate that differences in incidence and mortality between countries translate to wide age ranges at which comparable levels of risk are reached. Age-specific CRC incidence and mortality represent important parameters regarding potential benefits of screening, which have to be weighed against costs and potential adverse side effects when choosing the age at screening initiation. Our analyses suggest that the balance in favour of screening is likely to be reached at rather different ages in the various European countries.

The reasons for this variation are likely to be manifold. Differences in risk factor profiles, such as dietary habits, across European countries, may be most relevant for variation in CRC incidence. As regards mortality, major differences in survival of CRC patients between European countries also play an important role (Verdecchia *et al*, 2007).

Even though CRC incidence and mortality data are important, there are further, country-specific factors to be considered in the choice of the age range at which screening is offered. For example, an important parameter is remaining life expectancy (Ko and Sonnenberg, 2005). Furthermore, non-epidemiological criteria, such as the availability and costs of various screening modalities in different countries have to be taken into account.

Risk adapted variation of ages for CRC screening initiation is already well accepted and established for CRC risk factors other than ‘country’, in particular a family history of CRC (Europe Against Colorectal Cancer, 2007). The ‘risk advancement’ (Brenner *et al*, 1993) given a positive family history is of similar magnitude than the between country differences of up to 10 years or more observed in the present analyses (Brenner *et al*, 2008). As regards gender differences, risk advancement among men compared to women is roughly 5 years (Brenner *et al*, 2007). Taken together, these patterns suggest that appropriate differentiation of age at initiation of CRC screening by gender and country might be similarly or even more relevant from a public health point of view than the widely practised differentiation by family history, an important but relatively infrequent CRC risk factor.

In the interpretation of our results, the following limitations should be kept in mind. Our analyses are based on data on CRC incidence and mortality by age and sex. Additional factors, such as potential variation in screening effectiveness by age, for which data are lacking, were not considered. In some of the countries included in this analysis, some form of CRC screening has already been practised before 2002, but, because of mostly small regional coverage (Benson *et al*, 2008) it is unlikely to have had a major effect on CRC incidence and mortality on the national level. Although estimates on CRC incidence and mortality in 2002 obtained from GLOBOCAN 2002 are based on the best available data sources, they are subject to uncertainties due to lack of or incomplete population coverage of cancer registration in many countries.

Analyses were presented in detail for selected ‘threshold levels’ of incidence and mortality only. Latter were defined by median levels across countries among men at ages 50, 55 and 60 years, which may not necessarily be the best ‘threshold levels’. Best threshold levels and optimum age for commencing screening will be based on cost-benefit decisions. However, as can be seen from Figures 1 and 2, differences in ages at which thresholds are reached would be rather similar for any other intermediate levels of incidence and mortality within the assessed range.

In summary, our analyses do not allow deriving a general recommendation regarding the best age for initiation of CRC screening in each country. Our results do suggest, however, that the optimal age for screening initiation is likely not to be the same for European countries and that variation by up to 10 years or even more across countries might be warranted because of major differences in CRC incidence and mortality.

## Figures and Tables

**Table 1:** Age at which colorectal cancer incidence among men and women in European regions and countries reaches median values across countries for men at ages 50, 55, and 60 years (age_mi50_, age_mi55_, age_mi60_)

		**Men**			**Women**		
**Region**	**Country**	**age_mi50_**	**age_mi55_**	**age_mi60_**	**age_mi50_**	**age_mi55_**	**age_mi60_**
*East*	Belarus	51	57	62	52	62	71
	Bulgaria	51	57	63	55	64	72
	Czech Republic	45	**51 ↓**	**54 ↓**	50	55	61
	Hungary	**45 ↓**	51	54	**48 ↓**	55	61
	Moldova	48	55	62	52	61	75
	Poland	51	55	60	52	59	65
	Romania	52	59	66	56	**67 ↑**	**77 ↑**
	Russian Fed.	51	56	61	52	60	71
	Slovakia	46	51	54	50	57	63
	Ukraine	51	56	61	52	63	77
							
North	Denmark	50	55	60	50	57	62
	Estonia	52	57	61	53	60	65
	Finland	53	60	64	54	62	68
	Iceland	51	57	61	52	57	63
	Ireland	49	53	58	52	59	64
	Latvia	50	60	64	54	63	71
	Lithuania	52	57	62	55	64	72
	Norway	49	54	58	48	**54 ↓**	**60 ↓**
	Sweden	51	57	61	52	59	64
	United Kingdom	50	54	59	52	60	64
							
South	Albania	50	56	62	51	62	72
	Bosnia/Herzegovina	49	54	59	51	60	67
	Croatia	47	53	57	50	58	64
	Greece	**55 ↑**	**62 ↑**	**67 ↑**	**58 ↑**	65	72
	Italy	48	54	59	49	58	64
	Macedonia	51	57	62	53	62	69
	Malta	51	58	63	49	61	67
	Portugal	50	54	59	52	61	67
	Slovenia	48	53	56	51	58	64
	Spain	49	54	60	52	61	66
	Serbia/Montenegro	50	56	61	51	62	71
							
West	Austria	49	54	58	52	59	64
	Belgium	51	55	60	52	59	64
	France	48	54	58	51	59	65
	Germany	46	52	57	49	57	62
	Luxembourg	47	53	57	51	59	63
	Netherlands	49	54	58	50	56	62
	Switzerland	49	53	57	51	60	65

Lowest (↓) and highest (↑) ages in each column are marked bold.

**Table 2:** Age at which colorectal cancer mortality among men and women in European regions and countries reaches median values across countries for men at ages 50, 55, and 60 years (age_mm50_, age_mm55_, age_mm60_)

		**Men**			**Women**		
**Region**	**Country**	**age_mm50_**	**age_mm55_**	**age_mm60_**	**age_mm50_**	**age_mm55_**	**age_mm60_**
*East*	Belarus	49	54	58	50	59	64
	Bulgaria	47	54	60	50	60	65
	Czech Republic	44	51	53	48	56	61
	Hungary	**43 ↓**	**51 ↓**	**53 ↓**	46	**54 ↓**	**60 ↓**
	Moldova	45	53	58	50	58	66
	Poland	50	55	59	52	60	64
	Romania	50	57	62	52	62	68
	Russian Fed.	49	54	58	50	56	61
	Slovakia	45	51	53	49	56	61
	Ukraine	48	53	57	50	57	64
							
North	Denmark	48	54	59	48	58	61
	Estonia	51	55	60	51	58	63
	Finland	53	61	64	54	63	67
	Iceland	54	60	62	53	60	63
	Ireland	45	52	56	51	60	63
	Latvia	47	55	61	52	60	63
	Lithuania	50	55	59	52	60	64
	Norway	50	57	61	50	58	62
	Sweden	52	59	62	54	62	65
	United Kingdom	50	56	61	53	61	64
							
South	Albania	50	55	60	51	60	65
	Bosnia/Herzegovina	49	54	58	51	59	63
	Croatia	48	53	56	51	58	62
	Greece	**54 ↑**	**62 ↑**	**65 ↑**	**57 ↑**	**64 ↑**	**69 ↑**
	Italy	50	57	61	53	62	65
	Macedonia	51	59	63	53	63	68
	Malta	52	55	58	**45 ↓**	57	64
	Portugal	50	55	60	52	61	64
	Slovenia	47	53	56	50	60	63
	Spain	49	56	61	52	61	65
	Serbia/Montenegro	49	54	59	50	59	64
							
West	Austria	50	55	60	52	60	63
	Belgium	51	56	61	52	60	63
	France	51	57	61	53	61	65
	Germany	50	55	60	52	60	62
	Luxembourg	51	56	61	54	61	64
	Netherlands	50	56	60	51	60	63
	Switzerland	52	58	62	55	62	66

Lowest (↓) and highest (↑) ages in each column are marked bold.

## References

[bib1] Advisory Committee on Cancer Prevention (2000) Recommendations on screening in the European Union. Eur J Cancer 36: 1473–1478 10930794 10.1016/s0959-8049(00)00122-2

[bib2] Benson VS, Patnick J, Davies AK, Nadel MR, Smith RA, Atkin WS, on behalf of the International Colorectal Cancer Screening Network (2008) Colorectal cancer screening: a comparison of 35 initiatives in 17 countries. Int J Cancer 122: 1357–1367 18033685 10.1002/ijc.23273

[bib3] Brenner H, Gefeller O, Greenland S (1993) Risk and rate advancement periods as measures of exposure impact on the occurrence of chronic diseases. Epidemiology 4: 229–236 8512987 10.1097/00001648-199305000-00006

[bib4] Brenner H, Hoffmeister M, Arndt V, Haug U (2007) Gender differences in colorectal cancer: implications for age at initiation of screening. Brit J Cancer 96: 828–831 17311019 10.1038/sj.bjc.6603628 PMC2360074

[bib5] Brenner H, Hoffmeister M, Haug U (2008) Family history and age at initiation of colorectal cancer screening. Am J Gastroenterol (in press) 10.1111/j.1572-0241.2008.01978.x 18702651

[bib6] Europe against colorectal cancer (2007) Declaration of Brussels, http://www.future-health-2007.com/fileadmin/user_upload/Brussels_Declaration.pdf.accessed 4 April 2008

[bib7] Ferlay J, Autier P, Boniol M, Heanue M, Colombet M, Boyle P (2007) Estimates of the cancer incidence and mortality in Europe in 2006. Ann Oncol 18: 581–592 17287242 10.1093/annonc/mdl498

[bib8] Ferlay J, Bray F, Pisani P, Parkin DM, GLOBOCAN 2002 (2004) Cancer Incidence, Mortality and Prevalence Worldwide. IARC Cancer Base No. 5 Version 2.0. IARC Press: Lyon, France

[bib9] Ko CW, Sonnenberg A (2005) Comparing risks and benefits of colorectal cancer screening in elderly patients. Gastroenterology 129: 1163–1170 16230070 10.1053/j.gastro.2005.07.027

[bib10] Malila N, Anttila A, Hakama M (2005) Colorectal cancer screening in Finland: details of the national screening programme implemented in autumn 2004. J Med Screen 12: 28–32 15814016 10.1258/0969141053279095

[bib11] Parkin DM, Bray F, Ferlay J, Pisani P (2005) Global cancer statistics, 2002. CA Cancer J Clin 55: 74–108 15761078 10.3322/canjclin.55.2.74

[bib12] Pox C, Schmiegel W, Classen M (2007) Current status of screening colonoscopy in Europe and in the United States. Endoscopy 39: 168–173 17327977 10.1055/s-2007-966182

[bib13] United Nations Population Division (2007) World Population Prospects. The 2006 Revision. Population Database http://esa.un.org/unpp/, accessed 19 October 2007

[bib14] Verdecchia A, Francisci S, Brenner H, Gatta G, Micheli A, Mangone L, Kunkler I, the EUROCARE-4 Working Group (2007) Recent cancer survival in Europe: a 2000–02 period analysis of EUROCARE-4 data. Lancet Oncol 8: 784–796 17714993 10.1016/S1470-2045(07)70246-2

[bib15] West NJ, Poullis AP, Leicester RJ (2007) The NHS Bowel Cancer Screening Programme–a realistic approach with additional benefits. Colorectal Dis e-pub ahead of print 23 October 10.1111/j.1463-1318.2007.01396.x 17956587

